# Rapid Fabrication of Membrane-Integrated Thermoplastic Elastomer Microfluidic Devices

**DOI:** 10.3390/mi11080731

**Published:** 2020-07-28

**Authors:** Alexander H. McMillan, Emma K. Thomée, Alessandra Dellaquila, Hussam Nassman, Tatiana Segura, Sasha Cai Lesher-Pérez

**Affiliations:** 1 Elvesys Microfluidics Innovation Center, 75011 Paris, France; alexanderhsiaoyen.mcmillan@kuleuven.be (A.H.M.); thomee@etu.unistra.fr (E.K.T.); alessandra.dellaquila@uni-bielefeld.de (A.D.); 2 Centre for Membrane Separations, Adsorption, Catalysis and Spectroscopy for Sustainable Solutions (cMACS), Department of Microbial and Molecular Systems, KU Leuven, Celestijnenlaan 200F, 3001 Leuven, Belgium; 3 Université de Strasbourg, CNRS, UMR7140, 4 Rue Blaise Pascal, 67081 Strasbourg, France; 4 Biomolecular Photonics, Department of Physics, University of Bielefeld, 33615 Bielefeld, Germany; 5 Department of Biomedical Engineering, Duke University, Durham, NC 27708, USA; hussamynassman@gmail.com (H.N.); tatiana.segura@duke.edu (T.S.)

**Keywords:** rapid fabrication, thermoplastic elastomer, microfluidic device, membrane-based cell culture, delamination testing

## Abstract

Leveraging the advantageous material properties of recently developed soft thermoplastic elastomer materials, this work presents the facile and rapid fabrication of composite membrane-integrated microfluidic devices consisting of Flexdym^TM^ polymer and commercially available porous polycarbonate membranes. The three-layer devices can be fabricated in under 2.5 h, consisting of a 2-min hot embossing cycle, conformal contact between device layers and a low-temperature baking step. The strength of the Flexdym^TM^-polycarbonate seal was characterized using a specialized microfluidic delamination device and an automated pressure controller configuration, offering a standardized and high-throughput method of microfluidic burst testing. Given a minimum bonding distance of 200 μm, the materials showed bonding that reliably withstood pressures of 500 mbar and above, which is sufficient for most microfluidic cell culture applications. Bonding was also stable when subjected to long term pressurization (10 h) and repeated use (10,000 pressure cycles). Cell culture trials confirmed good cell adhesion and sustained culture of human dermal fibroblasts on a polycarbonate membrane inside the device channels over the course of one week. In comparison to existing porous membrane-based microfluidic platforms of this configuration, most often made of polydimethylsiloxane (PDMS), these devices offer a streamlined fabrication methodology with materials having favourable properties for cell culture applications and the potential for implementation in barrier model organ-on-chips.

## 1. Introduction

The choice of the materials used to create a microfluidic device is critical to its ultimate function. A given material should be evaluated from two perspectives—its material properties and its fabrication processes. The latter becomes particularly influential when complex device geometries are desired. An increasingly relevant example of this is porous membrane-integrated microfluidic devices for cell culture, whose non-trivial construction has been the attention of much research. The use of thin, porous membranes as a cell culture substrate has shown great value for studying cell-cell signalling, cell filtration and cell migration, in both static [1,2,3,4,5] and more recapitulative dynamic microfluidic models [6,7,8]. At the forefront of membrane-based cell culture is “organ-on-chip” (OOC) technology, which often consists of two adjacent compartments separated by a porous membrane. By culturing cells on both sides of the thin porous membrane, tissue-tissue-like interfaces can be generated that simulate critical physiological barriers, such as that of the blood-brain barrier [9], liver [10] and the epithelial-endothelial membranes in the lung [11], kidney [12] and gut [13], among other human organs and tissues [14,15,16].

While a variety of materials have been used, many of the cutting-edge microfluidic membrane-based cell culture platforms have been based around polydimethylsiloxane (PDMS) devices [17]. PDMS, the most ubiquitous microfluidic material for biology, has undoubtedly shaped the advancement of microfluidics since George Whitesides’ group introduced its soft lithography microfabrication techniques in 1998 [18]. Its acceptance as a standard material for microfluidics can be attributed to its favourable material properties of high optical clarity, biocompatibility and easy handling due to its elasticity and low stiffness (tensile modulus of ~1–3 MPa [19,20,21]). Furthermore, PDMS microfabrication could be achieved at relatively low cost and little required expertise as compared to other materials at that time.

PDMS suffers, however, from a number of drawbacks in its use as a microfluidic device substrate, the most prevalent of which are small molecule absorption, hydrophobic recovery and transferability of fabrication. (i) Absorption of small hydrophobic molecules into the bulk of the material [22] is problematic in applications that involve soluble factors, namely drug, cell signalling and dye compounds [23,24,25], where essential concentrations can be altered and experimental outcomes changed. This severely limits the utility of PDMS for drug screening, a key area of therapeutic research and development that membrane-based cell culture platforms can address. (ii) Fast hydrophobic recovery after surface hydrophilization due to mobile polymer chains [26] limits the shelf-life of PDMS devices post-fabrication, imposing a burden on the end-user to handle the hydrophilization, whereby devices must be experimentally used within hours of their preparation for effective surface treatments and channel filling. (iii) Finally, the poor transferability of fabrication, from small to large-scale, limits PDMS from greater industrial implementation. While PDMS allows relatively facile fabrication of microfluidic devices when compared to glass or silicon-based microdevices [27], its multi-step process involving liquid polymer mixing and degassing, curing, plasma bonding (or other less-traditional bonding techniques) and the aforementioned hydrophilization, does not lend itself well to being transferred to larger, industrial-scales [28]. Microfluidic models developed in labs using PDMS must thus be reimagined with different materials if large-scale implementation is to be feasible, a modification likely to have side-effects on experimental outcomes. This presents complications for encouraging the wider adoption of microfluidics to replace more conventional biological research methods in industry. Additionally, the fabrication of thin, porous PDMS membranes is time-consuming and intricate [29,30,31,32,33,34] and further hinders the reproducible high-throughput production of PDMS membrane platforms. As an alternative, the utilization of commercially available track-etched porous polymer membranes that are biocompatible and available in a range of material compositions, thicknesses (down to 7 µm [35]), pore sizes and porosities circumvents the custom fabrication of membranes for membrane-based cell culture devices [36].

Track-etched membranes reflect one aspect of a growing interest in thermoplastic microfluidic devices, which can not only address some of the material property concerns around PDMS but also leverage the wealth of industrial processing knowledge that exists for this class of materials for high throughput manufacturing [37]. The fact alone that the vast majority of current cell biology research is conducted on substrates of polystyrene (PS) should not be neglected when considering the forces at play in a shift toward greater adoption of thermoplastics for microfluidic techniques [38]. Hard thermoplastics, such as PS, polycarbonate (PC), polymethylmethacrylate (PMMA) and cyclic olefin copolymer (COC), are low cost materials that can be melt-processed with high-throughput techniques, namely injection moulding and hot embossing and have shown much promise and utility as microfluidic substrates [39,40,41,42,43]. These materials, however, largely due to their rigidity (of tensile moduli in the order of ~1–4 GPa [44]), entail difficulties in processing at small scales, including the need for expensive moulds and process-intensive bonding and interfacing to fluidic setups that make their use rather prohibitive to those without the sufficient means or fabrication expertise.

The introduction of a class of materials known as thermoplastic elastomers (TPE) or soft thermoplastic elastomers (sTPE), for microfluidics has provided for a unique combination of the rapid and high-throughput processing of thermoplastics with the flexible and easy handling of elastomers like PDMS [45,46,47,48]. One such commercially-available polymer called Flexdym^TM^ (FD) has been shown to have particularly advantageous material properties for its use as a microfluidic device substrate [49,50]. It is a soft (tensile modulus of ~1 MPa) and flexible styrenic block co-polymer that is biocompatible and optically transparent. It can be rapidly hot embossed with high resolution within minutes using microfluidic moulds that are simple and low-cost as compared to the moulds needed for moulding hard thermoplastics, which tend to be more expensive and require more complex fabrication. Thanks to its hard and soft block co-polymeric structure, FD has adhesive and cohesive bonding properties to allow for facile and spontaneous sealing of microfluidic devices after moulding without the need for additional adhesives or plasma surface treatment [49]. Indeed, FD has been described as a “slow” adhesive polymer foil and has been shown to create reversible bonds with itself and other polymer surfaces, which can be strengthened at elevated temperatures [49,51]. The sTPE has additionally demonstrated more stable hydrophilization with plasma treatment and lower absorption of small hydrophobic molecules as compared to PDMS [49]. This material very importantly offers the transferability of fabrication that both PDMS and hard thermoplastics lack; it permits rapid and accessible fabrication in research laboratory settings, while also providing a feasible scope for scaling up to industrial production. This transition can be made without altering the material and, very critically, any influence this may have on the test at hand.

In this work we present a composite microfluidic device based on the FD polymer and a commercially available porous polycarbonate membrane designed for use as a membrane-integrated cell culture platform. We developed a rapid and scalable fabrication protocol and characterized the bonding integrity that can be achieved as well as the flow characteristics in devices representing typical microfluidic cell culture geometries for a practical translation of device pressure capabilities. Finally, we confirmed that cell attachment and sustained cell adhesion and culturing was possible inside the devices, giving a proof-of-concept for a facile, robust and scalable microfluidic platform for membrane-based cell culture.

## 2. Materials and Methods 

### 2.1. Composite Device Microfabrication

#### 2.1.1. Mould Fabrication

Microfluidic moulds were fabricated using Ordyl^®^ SY 300 dry film negative photoresist (55 μm thickness, ElgaEurope s.r.l., Milan, Italy) on 75 mm × 50 mm borosilicate glass slides (Corning Inc., Corning, NY, USA). After cleaning with acetone and isopropanol and dehydration of the glass slide on a hotplate (Thermo Fisher Scientific, Waltham, MA, US) for 5 min at 150 °C, two sheets of photoresist were laminated onto the slide using a thermal laminator (325R6, FalconK, France) at 120 °C and roller speed 4. Using an exposure masking UV LED lamp (UV-KUB 2, Kloé, Montpellier, France) the photoresist was then exposed to UV light (365 nm, 23.3 mW/cm^2^), for 7 s with a film photomask (Selba S.A., Versoix, Switzerland) and subsequently developed with a solvent blend (Ordyl^®^ SY Developer, ElgaEurope s.r.l., Milan, Italy) for approximately 10 min to remove unexposed sections of the photoresist. The mould fabrication process was finished with a hard bake of 30 min at 120 °C on a hotplate. This mould can be used for both sTPE hot embossing as well as PDMS soft lithography. Moulds with thicker features can be achieved by laminating successive layers of the photoresist before the exposure masking step.

#### 2.1.2. Hot Embossing

**Figure 1:**
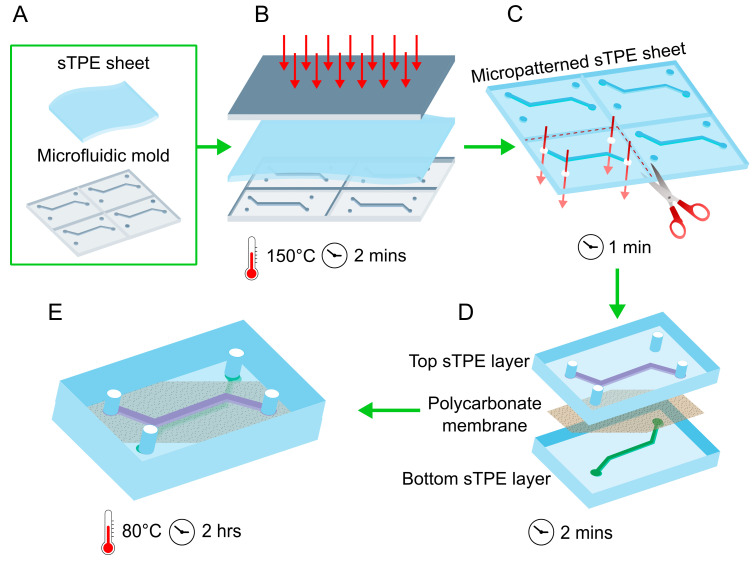
Schematics of the composite membrane-integrated cell culture device fabrication workflow starting from (**A**) a pre-extruded Flexdym^TM^ sTPE sheet and a microfluidic mould. Fabrication consists of (**B**) a 150 °C hot embossing cycle of the sTPE sheet atop a microfluidic mould, (**C**) cutting of the micropatterned sTPE to appropriate device size and punching access holes, (**D**) layering of the micropatterned sTPE layers with an off-the-shelf porous polycarbonate membrane and (**E**) baking at 80 °C to achieve device bonding resulting from the mobility of the intrinsically adhesive “soft” block polymer chains. Devices of this configuration used for cell culture contained channels of cross section 800 µm × 110 µm (width × height) and 27 mm length. The durations of each fabrication step are included.

Extruded sheets of FD polymer (Eden Microfluidics SAS, Paris, France) of 1.3 mm thickness were cut with scissors to approximately the size of the glass slide and cleaned with tape to remove any large dust particles (Figure 1A). They were then manually placed into contact with the photoresist features on the mould, ensuring good contact and minimal air bubbles between the FD sheet and the mould. A clean, blank glass slide was then similarly pressed into contact with the other side of the FD sheet and the entire assembly (mould-FD-glass slide) was placed in a vacuum-assisted heat press (Sublym100^TM^, Eden Microfluidics SAS, Paris, France) between two aluminium plates. The assembly was subjected to an isothermal hot embossing cycle of 2 min at 150 °C and 0.7 bar applied pressure, corresponding to approximately 6.5 bar of pressure on the stacked assembly. Spacers of 2.3 mm thickness were additionally placed in between the aluminium plates to control for a final FD thickness of 1.1 mm (Figure 1B). The assembly was removed and separated using isopropanol to facilitate separation of the hot embossed FD from the mould and glass slide. Four holes were punched in one sTPE sheet with a steel hole punch at the appropriate port locations and the resulting micropatterned FD could again be cut with scissors to the desired size before microfluidic device assembly (Figure 1C).

#### 2.1.3. Device Assembly and Bonding

The assembly of an FD-PC composite device was achieved by layering a porous track-etched polycarbonate membrane (2 μm pores, 5.6% porosity, 23 μm thickness, Isopore^TM^, Merck KGaA, Darmstadt, Germany) in conformal contact with the micropatterned side of the FD sheet, applying pressure with tweezers to ensure contact and avoid air bubbles. Light, reversible adhesion occurs immediately between the PC membrane and the sTPE sheet. The PC membrane was manually placed with tweezers on the sTPE layer such that it covered the entirety of the channel and its two access holes (top layer in Figure 1D) but left the remaining two holes unobstructed for access to the channel on the second sTPE layer (bottom layer in Figure 1D). The second micropatterned sheet of FD, with no holes punched, was then similarly layered manually with tweezers atop the PC membrane with the aid of a stereoscope to ensure proper channel alignment. The two central channels were in direct superposition and the second channel inlet and outlet aligned with the access holes punched in the first sTPE layer (Figure 1D). The light adhesion that occurs immediately upon placement of the second sTPE layer can easily be reversed, allowing for any poor alignment to easily be corrected. The device was then inverted such that the sTPE layer with access holes was on top (Figure 1E). This configuration represents a three-layer, two-channel device, where channel geometries exist on both sides of the membrane. Alternatively, the second FD sheet can be devoid of features in order to create a single-channel device; this variation will be discussed in further detail below in Section 2.2.1. 

Conical FD connectors (Eden Microfluidics SAS, Paris, France), to interface with microfluidic tubing (not shown in Figure 1), were fixed atop the device ports by first placing the connector on a silicon wafer on a hotplate at 150 °C for 10 s in order to achieve a smooth, flat surface, then immediately transferring it in contact with the FD substrate at the desired port location. This final assembly step can vary depending on the desired method of device interfacing and connection (such as compression or adhesive-based connectors). The entire FD-PC-FD assembly was then baked in a forced convection oven (DKN612C, Yamoto Scientific Co. Ltd., Tokyo, Japan) at 80 °C for 2 h to achieve bonding between the three layers (Figure 1E) without the need for plasma activation or adhesives, thanks to the intrinsic adhesive characteristics of the sTPE (described further in Section 3.1). The entire device fabrication process is summarized in Figure 1 and Figure S1 shows more detailed step-by-step images of the fabrication process. The same protocol can be followed, minus the addition of the polycarbonate membrane, to fabricate single or multi-channelled devices made entirely of FD, such as the devices made entirely of FD for delamination testing, as detailed further in Section 2.2.1.

### 2.2. Delamination Testing

#### 2.2.1. Delamination Device

**Figure 2:**
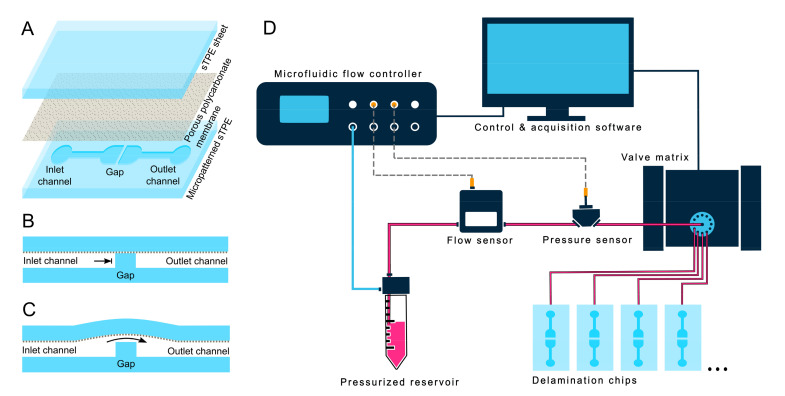
(**A**) Expanded view of the FD-PC-FD microfluidic chip design for delamination tests, consisting of two disconnected channels separated by a gap of varying distances. The inlet channel is increasingly pressurized, with no flow occurring until the delamination of the PC membrane from the FD gap structure occurs, at which point fluid crosses the gap into the outlet channel. (**B**) And (**C**) respectively show cross sections of the gap portion of the device before and after delamination. (**D**) Schematic of the automated delamination testing setup utilizing flow and pressure sensors and a valve matrix in series with a water-filled reservoir pressurized by a pressure controller. Continuous data logging and sensor feedback allowed the sequential testing of the pressure capacities of up to 10 microfluidic devices with no user monitoring.

The integrity of bonding between FD and the PC membrane, as well as between FD and FD substrates, was evaluated by using a device with two disconnected channels separated by varying gap distances (Figure 2A–C). A FD-PC-FD device (containing one micropatterned FD sheet and one featureless FD sheet, separated by a PC membrane) was fabricated with a mould of this channel-gap design. When pressure was applied to the input, no fluid could flow except in cases where delamination across the gap occurred, that is, the PC and FD bonded at the gap separated and allowed for the passage of fluid from the input to the output channel.

#### 2.2.2. Automated Delamination Testing

FD-PC delamination devices were tested with a microfluidic setup (Figure 2D) consisting of an OB1^®^ MK3+ pressure controller (0–2000 ± 0.1 mbar), thermal flow sensor (MFS3, -0–80 μL/min ± 5% m.v.) and capillary pressure sensor (MPS3, -1000–2000 ± 6 mbar), where pressure was applied from the pressure controller and transmitted to the device via water in a reservoir and polytetrafluoroethylene (PTFE) microfluidic tubing (all microfluidic equipment from Elveflow^®^, Elvesys SAS, Paris, France). Delamination devices were connected ensuring that no bubbles were present in the microfluidic system. A stepwise pressure profile between 0 and 2000 mbar gauge pressure, with 50 mbar steps lasting 30 s each, was executed using the Elveflow^®^ Smart Interface software. The pressure controller interface logged the in-line flow and pressure sensor data and was programmed to stop the pressure sequence if a leak was detected. Such a leak was indicated by a sudden increase to a non-zero flow rate and drop in pressure at the device inlet. A valve multiplexer (MUX Distributor) allowed for the sequential testing of up to ten devices in a single program execution.

This synchronized logging of data from both the sensors as well as the pressure controller itself offered redundancy to reduce erroneous results and allowed for the precise confirmation of the moment and pressure at which delamination between the FD and PC occurred. By using a single software interface for both data logging and equipment control, feedback loops could be straightforwardly implemented to cut a testing cycle short as soon as a delamination event was detected and subsequently switch devices.

Delamination devices with gap distances between 100 and 1000 μm were tested in this manner (n = 5 per gap distance) to evaluate the effect of the bonding distance on the resulting FD-PC bond strength. Delamination tests were repeated on a set of devices lacking PC membranes, for comparison of FD-PC bond strength with that of FD-FD self-bonding.

To investigate the stability of device bonding over time in order to simulate long-term cell culture and repetitive use, similar pressure delamination tests were conducted on devices of 400 μm gap distance at different time points after fabrication (1, 7 and 14 days post fabrication). Devices were aged at either room temperature or in an incubator (Model H2200-H, Benchmark Scientific Inc., Sayreville, NJ, USA) at 37 °C and high humidity to simulate cell culture conditions. Statistical analysis consisted of a one-way analysis of variance (ANOVA) between the six FD-PC delamination groups to evaluate if time and incubation resulted in a statistically significant difference in delamination pressure of FD-PC devices, where variation was considered significant when *p* < 0.05. 

Device stability under long term pressure conditions was tested with devices in the same delamination setup both with static and cyclic pressures to evaluate the device robustness and durability. Static tests were conducted by pressurizing the devices to 500 mbar for a period of 10 h (n = 5) and cyclic tests by subjecting devices to 10,000 cycles of 0 to 500 mbar pressure at 0.2 Hz (n = 5). 

### 2.3. Flow Evaluation

Flow tests were conducted on FD-PC devices consisting of a simple channel of 27 mm length, 55 μm height and varying width (200, 400, 800 μm) atop a PC membrane and second sheet of un-patterned FD. This design was a single-channel version of the two-channel device represented in Figure 1E. The microfluidic circuit consisted of (i) approximately 50 cm of 0.8 mm inner-diameter (ID) PTFE tubing; (ii) a flow sensor with a quartz capillary of 430 μm ID and 3 cm in length (MFS3, -80–80 μL/min ± 5% m.v.); (iii) a capillary pressure sensor with an effective ID of 0.8 mm and length of 8 mm (MPS3, -1000–2000 ± 6 mbar); (iv) the microfluidic channel; and (v) a 5 cm section of polyether ether ketone (PEEK) tubing of 120 μm ID. The PEEK tubing was inserted into the microfluidic circuit downstream from the chip for added microfluidic resistance to simulate additional components in the system. Pressure and flow rate data were collected across the microfluidic setup (n = 3 devices per channel size) and corresponding fluid shear stresses experienced on the PC membrane surface were calculated in order to provide an evaluation of the fluid mechanical conditions achievable within the pressure range that the composite devices can withstand. 

### 2.4. Cell Evaluation

Three-layer devices (see Figure 1E) were fabricated to have two chambers separated by a PC membrane, with each chamber having a cross section of 800 µm × 110 µm (width × height) and 27 mm length. These devices were UV-sterilized prior to any cell culture work. After sterilization, devices were pre-treated with plasma (BD-20AC laboratory corona treater, Electro-Technic Products, Chicago, IL, US) for 10 s to increase hydrophilicity of the membranes prior to incubating the devices with 10 µg/mL fibronectin (MilliporeSigma, Burlington, MA, USA) for 1 h at 37 °C. After fibronectin incubation, devices were flushed with 1X phosphate buffered saline (PBS) supplemented with 1% penicillin/streptomycin (Gibco^®^, Thermo Fisher Scientific). The upper channel was then loaded by pipette with 7 µL of human dermal fibroblasts (HDFs) (ATCC, Manassas, VA, USA) at a concentration of 2 × 10^5^ cells/mL in Dulbecco’s Modified Eagle Medium (DMEM) (high glucose, GlutaMAX^TM^ supplement, Thermo Fisher Scientific, Waltham, MA, USA) supplemented with 10% FBS (Corning Inc., Corning, NY, USA) and 1% penicillin/streptomycin. Cells were initially cultured for 12 h atop the PC membrane prior to exchanging media by flow to remove non-adhered cells. Calcein AM (Sigma-Aldrich, St. Louis, MO, USA) was applied to cells after 48 h of culturing in the devices by supplementing Calcein AM at 4 µM in 1X PBS for 20 min. Cells were imaged (Zeiss Observer Z1, Carl Zeiss AG, Oberkochen, Germany) after Calcein AM treatment, to verify the presence and distribution of cells in devices. Imaging was similarly repeated at 7 days after seeding. 

Cell fixing and staining with Alexa Fluor^TM^ 488 Phalloidin (Thermo Fisher Scientific, Waltham, MA, USA) and DAPI (Sigma-Aldrich, St. Louis, MO, USA) was done after 7 days of culturing cells in the upper channel of the devices. Briefly, cells were washed with PBS, treated with 4% paraformaldehyde (PFA) for 15 min at room temperature and then washed three times with PBS. Cells were then permeabilized with 0.3% Triton-X (Sigma-Aldrich, St. Louis, MO, USA) in PBS. Cells were subsequently stained with 488 Phalloidin and DAPI at 0.66 µM and 1 µg/mL, respectively, in PBS for 30 min prior to rinsing with PBS and imaging (Nikon C2 Confocal, Nikon, Tokyo, Japan).

## 3. Results and Discussion

### 3.1. Composite Device Microfabrication

Through vacuum-assisted isothermal hot embossing, FD sheets were patterned with microfluidic channels in 2 min. It is a moulding technique that is highly compatible with the already existing soft lithography expertise that is widespread in microfluidics labs, as there is no need for a specialized master mould; moulds that are commonly used for PDMS micropatterning, namely those derived from SU-8 [49], epoxy [49] and dry film photoresists (such as the Ordyl^®^ mould used in this work) can also be used for sTPE hot embossing. 

Hot embossing was followed by punching of ports then layering of subsequent PC and FD device layers in conformal contact. The soft, flexible properties of the sTPE allow for facile punching and readily achievable conformal contact, which can be both reproducibly completed in a matter of minutes (depending on the complexity of multi-layer devices requiring alignment), with little training. The co-polymeric properties of the materials allow for a reversible bond to be formed, while avoiding the necessity of adhesives or plasma activation of surfaces that are usually associated with polymeric microfluidic device sealing. This bonding results from macro-molecular motion of the sTPE’s ethylene-butylene (EB) soft polymer portion. The EB block possesses a negative glass transition temperature, allowing polymer chain mobility that can be promoted at elevated temperatures to facilitate spontaneous bonding with itself and other materials [49,52]. Full material and microstructure deformation is inhibited, however, by the PS hard block portion of FD, whose glass transition temperature remains above the baking temperature. This streamlines the process and simplifies any bonding optimization that may be required. Finally, the baking at 80 °C for 2 h is the most time intensive step in the fabrication process, however, baking time and temperature could be modified depending on the bonding strength required for specific device applications. Figure S2 shows a completed composite device.

From start to finish, beginning with the moulding procedure, the developed fabrication protocol results in devices in under 2.5 h. This presents a significant improvement on the production time of a comparable three-layer PDMS porous membrane device and the time savings are multiplied when the prospect of fabricating numerous devices is considered, a ubiquitous necessity for cell biology applications. In addition, a single master mould can be used to fabricate multiple devices in parallel, since it is only needed for the 2-min hot embossing channel formation step. PDMS, on the other hand, relies on relatively slow curing of its base polymer-crosslinker mixture, demanding that a single mould be in use for the entirety of the most time-intensive phase of fabrication, typically requiring between 1 to 4 h with baking or 48 h at room temperature [21,53].

The Isopore^TM^ membranes used in this study represent a readily available and inexpensive option within this class of track-etched polymeric membranes. Similar PC membranes have been effectively used in microfluidic cell culture studies and indeed for OOC applications [9,54,55,56,57,58]. The membranes are structurally robust, not requiring special handling techniques and their interaction with FD very crucially retains the spontaneous sealing property that the sTPE has with itself, allowing uncomplicated interfacing of composite layers. Thin porous membranes in literature, central to barrier model platforms, are often made of PDMS, requiring diverse and often complicated processes that are limited in their accessibility, reproducibility and ability to be high throughput. In combination with extruded FD sheets, which can similarly be stored and employed off-the-shelf, the PC membranes allow for rapid full-device fabrication with minimal time investment and planning that contrasts from PDMS methods. While the ability to elastically stretch the PC membranes was not evaluated, the mechanical properties of PC would suggest difficulty in achieving this at scales relevant to cellular mechanical stimuli. This presents a limitation when mechanical actuation is of greater significance and more elastic materials would be desirable, such as when modelling the alveolar interface in lung-on-chip systems [11]. Another potential drawback of these track-etched membranes is their micro-scale thickness, which can limit bright field imaging (discussed further in Section 3.4) and cell-cell juxtracrine signalling [35,59,60]. More recent advances in ultra-thin nano-scale membranes have shown improved optical clarity, permeability and cell contact [61] but they have yet to be made readily available for widespread implementation.

The fabrication of these composite devices represents a highly accessible, yet transferrable process. It leverages the elastomeric properties of sTPE materials for facile and inexpensive production at small lab-scales that shares equipment and know-how from soft lithography techniques (only requiring the addition of a heat press), while at the same time being higher throughput than PDMS production. Moreover, the thermoplastic nature of FD as well as the simplicity of fabrication steps gives scope for the scaling up of the developed fabrication protocol. Injection moulding or roll to roll hot embossing can be envisioned for the fabrication of large quantities of highly reproducible devices after prototyping and development at small research-scales, but, critically, using the same materials in both settings. This transferability between lab and industrial-scale is in sharp contrast to both PDMS and hard thermoplastic microfluidics (elaborated upon in the Introduction section).

### 3.2. Material Bonding Characterization

#### 3.2.1. Automated Delamination Testing

We developed an automated pressure testing setup to characterize the bonding strength between FD and PC membranes in a robust and precise manner. The developed setup allowed the sequential testing of up to ten samples with no user monitoring, regulated by feedback from continuous logging of pressure and flow rate data (Figure S3). This allowed a streamlined process of burst testing, reducing clean-up, observation and total time of experimentation required. Testing could be parallelized with the employment of multiple pressure and flow sensors for higher-throughput testing but the setup that was developed needs only two sensors, balancing speed with cost and practicality. 

Additionally, by varying the gap distance of the delamination device itself, the bonding characteristics of small features inherent to microfluidics could be investigated. This is significant in understanding the minimum feature sizes attainable with given materials in cases where, for example, thin channel walls or micro pillars are desired. 

A method of effectively sealing a microfluidic device is an integral part of its design and implementation and remains a continual challenge faced by the microfluidics community in the evaluation of new materials [62]. Leak/burst testing thus becomes imperative in assessing sealing techniques. Accordingly, while no standardized method specific to microfluidic applications exists, a wide variety of bond testing techniques have been used. This includes flow rate-based evaluation in flow-through channels and the pressurization of closed channel structures, both of which often rely on optical detection of leaks [63,64,65,66,67,68,69,70,71,72]. 

In comparison to the automated system developed here, these existing methods of burst testing remain low-throughput and examine the leaking of devices from a channel structure toward the exterior of the device in its entirety, often representing millimetres or centimetres of bonding distance (that is, the distance of bonded material that must delaminate for a leak/burst to occur). They do not consider the dynamics of delamination on smaller scales, which alter both small features and overall channel geometries and inevitably occur sooner than delamination of the device in its entirety. In this work, we thus propose a technique to test bonding that is both more representative in a microfluidic context and higher-throughput than existing methods, two aspects that will be vital in the future development and evaluation of new materials for microfluidic devices. 

#### 3.2.2. Flexdym^TM^-polycarbonate Bonding Strength

Balancing the integrity of a material bond with how easily it can be created is an engineering challenge in microfluidics that is highly dependent on the application at hand; the pressure capacity of devices made for cell culture will not be the same as that of devices made to handle supercritical fluids. We thus carried out delamination testing to evaluate the suitability of the composite FD-PC devices in the context of their utility for cell culture applications. More specifically, by using the above-described gap-channel delamination device, we investigated the minimum bonding distance that could be attained with the fabrication protocol developed in order to achieve sufficient and reliable bonding. This reproducibility in novel device development is something that is not often discussed but is vital in the realization of a robust microfluidic platform and the evaluation of its usability. 

**Figure 3:**
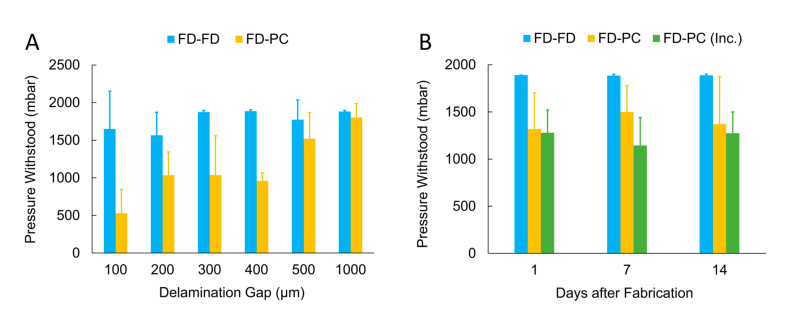
(**A**) FD-PC and FD-FD bonding evaluation through pressure delamination testing of devices with gap distances from 100 to 1000 µm. FD-PC devices show reduced bonding strength compared to FD-FD bonding but reliably withstand pressures of 500 mbar at gap distances of 200 µm and above. (**B**) Pressure delamination testing of FD-FD and FD-PC devices (fixed 400 µm gap distance) at 1, 7 and 14 days after fabrication. An additional set of FD-PC devices was aged in high humidity, 37 °C incubation conditions (abbreviated “Inc.” in the graph), which revealed no significant impact on the device sealing due to time post-fabrication or incubation conditions (n = 5 devices per dataset; error bars represent one standard deviation).

The pressure capacities of delamination devices (Figure 3A) investigating the FD-PC bond show an increase from 529 ± 318 mbar with a gap distance of 100 µm to 1802 ± 186 mbar with a gap distance of 1000 µm (noting that a maximum testing pressure of 2000 mbar was used, which, accounting for some pressure drop between the pressure controller and the devices, corresponded to a maximum pressure of ~1880 measured at the devices). This positive trend is characterized by high variability throughout the range of gap distances tested. The FD-FD devices show an overall increase in pressure capacity to ~1500 mbar and above at all gap distances. At gap distances of 300 µm and above the pressure capacity consistently corresponds with the bulk pressure capacity found by Lachaux et al. using a similar bonding protocol [49]. It is critical to note that an increased variability was also apparent at FD-FD gap distances of 100 and 200 µm. This could indicate a limitation of the manual process using tweezers to ensure reliable conformal contact at the gap when small dimensions are concerned. One potential way to minimize this variation would be through the use of microscope-assisted or automated procedures for more precision when creating conformal contact but would require more time invested per device. Minor spontaneous resealing of gap devices was observed after delamination occurred and device pressurization was released, without an additional baking step. Further characterization of FD-PC resealing was outside the scope of this work, as the focus was on microfluidic devices for cell culture, in which single-use devices are common practice. However, this phenomenon could prove to be interesting in other applications, such as normally-closed valves responding to varying pressure profiles, like those seen in microfluidic circuits and logic [73,74].

The superior pressure capacity of FD-FD devices as compared to FD-PC devices likely indicates a greater material interaction of FD with itself than with PC, as the bonding mechanism of such styrenic block copolymers relies on the mobility of EB polymer chains at the interface of the two like surfaces in contact [52]. It then follows that the PC, which does not contain the same EB blocks, has a weaker interaction with FD. Furthermore, PC has a higher glass transition temperature of ~150 °C that is not reached in the bonding procedure, which could result in reduced interaction due to polymer chain immobility. The reduced bonding strength could additionally underline lesser contact between the FD and PC surface as compared to FD-FD contact, which is facilitated due to the elastomeric properties of both device layers. Any unreliable contact would be accentuated at smaller scales and is indeed evident in the variability of FD-PC bonding at smaller gap distances, as well as in that of FD-FD. 

Nevertheless, at a bonding distance of 1 mm, a distance more representative of the milli-scale dimensions that typically define the material bond that seals a channel from its external environment, FD-PC devices frequently withstood maximum testing pressures. This quality of bonding at larger distances would characterize channels that do not contain thin separating walls or micro-scale structures and is more analogous to results reported using previously reported methods of bulk microfluidic burst testing [63,64,65,66,67,68,70,71,72]. Despite reduced bonding performance of FD-PC compared to FD-FD, at gap distances of 200 µm and above, FD-PC devices reliably withstood pressures of 500 mbar and greater, pressures that are generally sufficient for cell culture applications. The suitability of FD-PC device capacities in the context of their use for cell culture is discussed further in Section 3.3. 

While PDMS membrane-integrated cell culture systems have not expressly characterized the bond strength between the porous membrane and the rest of the device, most platforms of this type utilize oxygen plasma bonding between the PDMS slabs and the PDMS membrane [11,13,29,30,32]. Thus, the closest analog to FD-PC delamination data may be found in burst testing conducted in PDMS-PDMS plasma bonded systems. These PDMS-PDMS, covalent Si-O-Si, bonds are generally stronger than those exhibited by the FD-PC system, most often withstanding pressures between 2 and 3 bar [72,75] but are highly dependent on oxygen plasma parameters and have been reported ranging from approximately 0.7 to 4 bar [71]. In contrast, PDMS-PDMS sealing based only on conformal contact (without plasma surface activation) has been shown to leak at pressures above ~400 mbar [75]. Additionally, PDMS devices that use thermoplastic membranes, in a similar “sandwiched” configuration, primarily use a PDMS glue/mortar method [63] or chemical surface modification for covalent bonding [76]. These methods result in crosslinked or covalent bonds more representative of the PDMS to PDMS bonding strength, with maximum burst pressures of 1–1.2 bar for PDMS mortar and 2.27 bar for chemical bonding.

A complementary set of delamination tests were performed using devices of 400 µm gap distance with and without incubation at 37 °C and high humidity (similar to cell incubation conditions) for up to 14 days to investigate any bonding degradation that could occur resulting from the increased temperature and humidity conditions representative of the cell culture applications envisioned (Figure 3B). Only one gap distance was used for these tests, which were aimed at evaluating uniquely the effects of time and incubation-like conditions. 400 µm devices were chosen, as they were found to be the largest gap size that consistently delaminated within the test pressure range. After 14 days in incubation conditions, FD-PC devices withstood pressures of 1274 ± 225 mbar, as compared to FD-PC devices tested one day after fabrication, which withstood pressures of 1280 ± 241 mbar and 1319 ± 382 mbar, with and without incubation conditions, respectively. This testing revealed no significant difference in the integrity of the FD-PC bond resulting from time after fabrication or exposure to cell culture conditions bond (ANOVA: F(5, 24) = 0.61, *p* = 0.69), indicating the suitability of such devices for long term cell culture studies.

To further evaluate the quality of bonding of the composite devices in a manner relevant to cell culture applications, we investigated the bonding performance of FD and PC with pressurization over periods longer than the 20-min pressure cycle discussed thus far. Long-term fluid perfusion across cell cultures for continuous transport of nutrients, waste and soluble factors has long been cited as one of the numerous advantages of studying cells on microfluidic platforms [77]. Thus, bonding behaviour under the influence of constant pressure for extended time periods, in addition to cyclic pressures, is critical to understanding the effectiveness and longevity of these devices. Devices of 400 µm gap distance showed no delamination resulting from pressurization at 500 mbar for 10 h, nor at cyclic pressurization (0 to 500 mbar, 0.2 Hz, 10,000 cycles), demonstrating robust and reproducible performance under realistic working conditions.

### 3.3. Flow-Pressure Correlation

The influence of shear stress on cells is a significant factor that must be considered in the attempt to recapitulate in vivo conditions inside of a microfluidic device. It has been shown to have a major impact on cell differentiation and ultimate function, such as drug metabolism and cytokine secretion, in various cell types from across the body [78,79,80]. Thus, the ability to implement and control the appropriate shear stresses on a cell population is a central enabling characteristic of microfluidic technology and a consideration that must be made at the design and fabrication stage of device development. With this in mind, flow tests of FD-PC composite devices were conducted to understand the flow rates and calculate the shear stresses attainable inside of our devices, serving as a contextualization of the device pressure capacity results obtained through delamination testing. A design consisting of a simple channel of varying widths atop a PC membrane was used as a model to represent geometries and flow characteristics present in typical barrier model cell culture chambers in literature in which there is no flow across the membrane, most notably models developed by Harvard University’s Wyss Institute [11,12,81,82].

**Figure 4:**
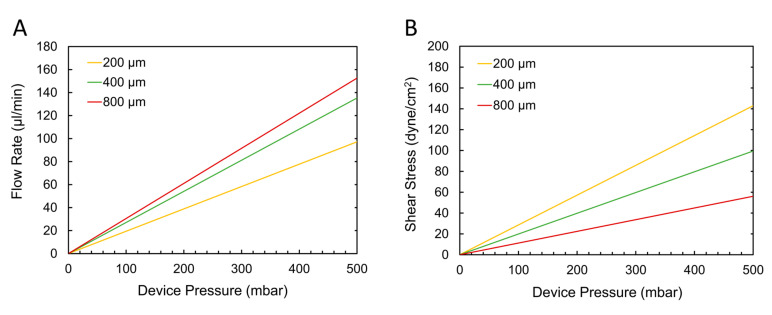
(**A**) Flow-pressure correlation in FD-PC devices from tests measuring the flow rate in a straight microfluidic channel (of width 200, 400 or 800 μm) and the corresponding pressure at the channel inlet. Within 500 mbar of pressure applied at the device, flow rates of up to approximately 150 μL/min can be reached. (**B**) Wall shear stresses that can be achieved in each of the example devices, as calculated from the flow rate data in (**A**), depending on the pressure applied. Shear stresses of up to approximately 140 dyne/cm^2^ can be generated with pressures of 500 mbar and below.

Figure 4A shows the linear relationships between the pressure measured at the inlet of the device and the flow rates in the given microfluidic setup and Figure 4B shows the corresponding shear stresses imposed on the surface of the membrane, as determined by the following equation describing the wall shear stresses, $\tau_{w}$, of laminar Newtonian fluids in a closed rectangular geometry:

(1) $$\tau_{w}=\frac{6\mu Q}{bh^{2}}\;,$$

where μ is the dynamic viscosity of the fluid (water, 8.90 × 10^−4^ Pa∙s at 25 °C), Q is the fluid flow rate, b is the channel width and h is the channel height [83]. This approximation of wall shear stress assumes parabolic Poiseuille flow in the microchannel, useful for estimating wall shear stresses in rectangular channels when flow is along the length of the channel and w > h. Depending on the channel dimensions used, flow rates of up to ~150 µL/min and shear stresses of up to ~140 dyne/cm^2^ could be achieved using 500 mbar or less of pressure applied to the composite devices. This gives ample range of control over fluid conditions inside the device and is sufficient for the shear stresses desired for *in vivo*-like cell culture conditions, which rarely surpass 25 dyne/cm^2^ [84]. The relatively low pressures required for such applications indicate that the FD-PC bonding strength discussed in Section 3.2.2, even with the presence of small features, would be sufficient for cell culture applications.

It must be noted that these relationships are dependent on the microfluidic resistance of the entire microfluidic system, which will inevitably vary from experiment to experiment, depending on the type and amount of devices, instruments and tubing that are being used. The introduction of a section of high-resistance PEEK tubing in the experimental flow setup downstream from the FD-PC devices served to simulate additional resistance that may exist in a setup and thus provide a conservative estimate of what pressures would be required to achieve a given flow rate. These results provide an aid in translating the pressure-based delamination findings into a more practically useful context (many microfluidic cell culture experiments depend on defining fluid flow rates or shear stresses rather than pressures) in order to assist potential users in understanding the capabilities of these devices.

### 3.4. Cell Culture

sTPE microfluidic devices have been used for cell culture in microfluidics [47,51], however, there has been limited published data associated with FD and its implementation in cell culture systems. To our knowledge, two different FD formulations have been previously reported in only two instances with cell culture work—(i) a mouldable film formulation of FD, similar to the one used in this study and (ii) a spin-coating formulation, FlexdymSC. The first showed cultured yeast cells [49] while demonstrating reduced absorption of a chemical division inhibitor due to FD’s material properties and FlexdymSC was shown to sustain culture of endothelial progenitor cells over four days [85]. Due to the limited published literature on culturing cells within FD microfluidic devices, we wanted to ensure that cultured cells could be maintained within our composite devices. To this end we cultured HDFs within our devices for one week.

**Figure 5:**
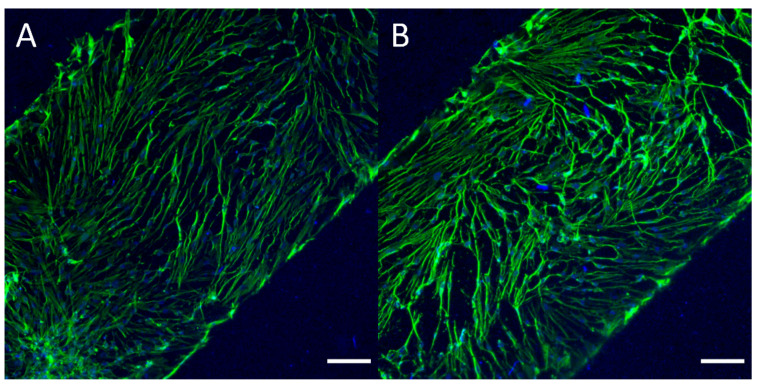
(**A**,**B**) Representative images of human dermal fibroblasts (HDFs) cultured in FD-PC-FD devices over the course of 7 days. HDFs presented a primarily spindle geometry, commonly seen when HDFs are cultured to high confluency, due to higher density of cells. HDFs were cultured on top of the polycarbonate membrane for 7 days prior to being fixed and stained with 488-Alexa Fluor^TM^ 488 Phalloidin (staining for F-actin, green) and DAPI (nuclear, blue), to demonstrate cell adhesion and maintained presence in static culture within devices over the course of 1 week. Scale bars = 150 μm.

HDFs were cultured in the FD-PC-FD microfluidic devices, with cells being seeded on the top of the polycarbonate membrane in the devices’ upper channels. We observed sustained cell adhesion and spread morphologies when cultured for up to one week (Figure S4). Additionally, cells were fixed and stained to visualize actin structures in cultured cells (Figure 5). The thickness of the polycarbonate membranes resulted in some difficulty in observing the cells under bright field illumination but did not pose a problem for fluorescent imaging.

While providing perfusion may be optimal to prime and stimulate more uniform cell alignment, proliferation and confluency throughout the microfluidic device, we wished to verify principally that the material and device configuration could sustain cells over multiple days. This was particularly of interest as sTPE materials similar to FD are known to have one to two orders of magnitude lower oxygen permeability than that of PDMS [86,87]. Static culturing with media exchanges every other day established that cells maintained good adhesion with spread morphologies over a one-week period within these devices without the need for more frequent perfusion. This demonstration, while limited in evaluating biological function, earmarks the potential use of this material and device configuration for barrier-like cell culture systems.

### 3.5. Drawbacks Compared to PDMS

When compared to three-layer, membrane-integrated PDMS microfluidic devices, our composite sTPE system has a few notable drawbacks. (i) The PC membranes have higher thickness and stiffness in comparison to porous PDMS membranes in the literature [35,59]. The more significant thickness of the thermoplastic membranes and their material properties reduces optical clarity, notably for bright field observation. Additionally, the diffusion and cell-cell contact, from one side of the membrane to the other, are reduced due the increased distance [60,61]. Furthermore, the non-elastomeric properties of the PC membrane largely prohibit membrane stretching to impose mechanical stresses on cell cultures, similar to those used in certain organ-on-chip devices [11,82]. (ii) Micropatterned sTPE sheets, in this and previous studies, are rather thin substrates, measuring ~1 mm in thickness, which limits the ability to define the final device thickness. This can introduce complications when interfacing microfluidic tubing with the device, requiring an additional connector solution. While numerous connector solutions exist, such as the conical sTPE connectors used in this work, this represents an additional fabrication step to use the sTPE device in a microfluidic setup. PDMS devices, on the other hand, can simply be fabricated with sufficient thickness to interface tubing directly into an access port thanks to its elastomeric properties. (iii) Styrenic block copolymer sTPE materials, like Flexdym^TM^, are known to have significantly lower oxygen permeability than PDMS [86,87]. While this did not pose problems for culturing cells in this work, this will result in a very different passive gas exchange and could potentially present difficulties in certain device geometries or flow regimes, requiring the user to incorporate a more involved gas control protocol to maintain appropriate oxygen levels inside a device.

## 4. Conclusions

Using the sTPE Flexdym^TM^ and a commercially available porous polycarbonate membrane, we have developed a composite microfluidic platform that can be fabricated in under 2.5 h with rapid hot embossing and facile self-sealing. The microfluidic devices consist of a membrane-separated chamber, similar to the geometries of membrane-based cell culture platforms in literature.

The bonding integrity of the devices was evaluated by testing the bond formed between the FD substrate and the PC membrane using an automated pressure delamination system to reproducibly test microfluidic material bonding in a high-throughput manner. FD-PC bond strength reliably withstood pressures of 500 mbar at bonding distances of 200 μm and greater, a pressure capacity that is largely sufficient for the needs of cell culture applications. The suitability of devices for cell culture was further highlighted by confirming no degradation of bonding strength in cell culture-like conditions and long-term pressurization. Finally, cell trials of HDFs showed good cell adhesion and a maintained culture atop PC membranes inside of composite devices over the course of one week, demonstrating the potential of these devices to be used for more extensive microfluidic cell culture models.

The promise that microfluidic cell culture technology offers in the advancement of in vitro platforms for drug testing and disease modelling has been tempered by the drawbacks of PDMS and the subsequent need for novel material solutions [88]. Our work introduces a microfluidic platform combining two materials with proven efficacy for cell culture research with a fabrication methodology that represents a rapid, facile and transferable solution.

## Supplementary Materials

The following are available online at https://www.mdpi.com/2072-666X/11/8/731/s1, Figure S1: Step-by-step fabrication procedure of a three layer, membrane-integrated sTPE-PC composite device; Figure S2: Composite device consisting of two micropatterned layers of Flexdym^TM^ sTPE separated by a porous polycarbonate membrane; Figure S3: Logic flowchart of the automated delamination testing setup programmed in the Elveflow Smart Interface software; Figure S4: Human dermal fibroblasts cultured in FD-PC-FD devices.
