# Supplementary material for: Rapid Fabrication of Membrane-Integrated Thermoplastic Elastomer Microfluidic Devices
Source: Micromachines (Basel). 2020 Jul 28;11(8):731. doi: 10.3390/mi11080731 (PMC7463978; doi:10.3390/mi11080731)
Supplement: Supplementary file 1 [file micromachines-11-00731-s001.pdf]

Supplementary Materials

Rapid Fabrication of Membrane-Integrated Thermoplastic Elastomer Microfluidic Devices

Alexander H. McMillan [1,2], Emma K. Thomée [1,3], Alessandra Dellaquila [1,4], Hussam Nassman [5], Tatiana Segura [5] and Sasha Cai Lesher-Pérez [1,*]

[1] Elvesys Microfluidics Innovation Center, 75011 Paris, France; alex.mcmillanl@elvesys.com
[2] Centre for Membrane Separations, Adsorption, Catalysis and Spectroscopy for Sustainable Solutions (cMACS), Department of Microbial and Molecular Systems, KU Leuven, Celestijnenlaan 200F, 3001 Leuven, Belgium; alexanderhsiaoyen.mcmillan@kuleuven.be
[3] Université de Strasbourg, CNRS, UMR7140, 4 Rue Blaise Pascal, 67081 Strasbourg, France; thomee@etu.unistra.fr
[4] Biomolecular Photonics, Department of Physics, University of Bielefeld, 33615 Bielefeld, Germany; alessandra.dellaquila@uni-bielefeld.de
[5] Department of Biomedical Engineering, Duke University, Durham, NC, USA; tatiana.segura@duke.edu
* Correspondence: sashacai.lesherperez@gmail.com

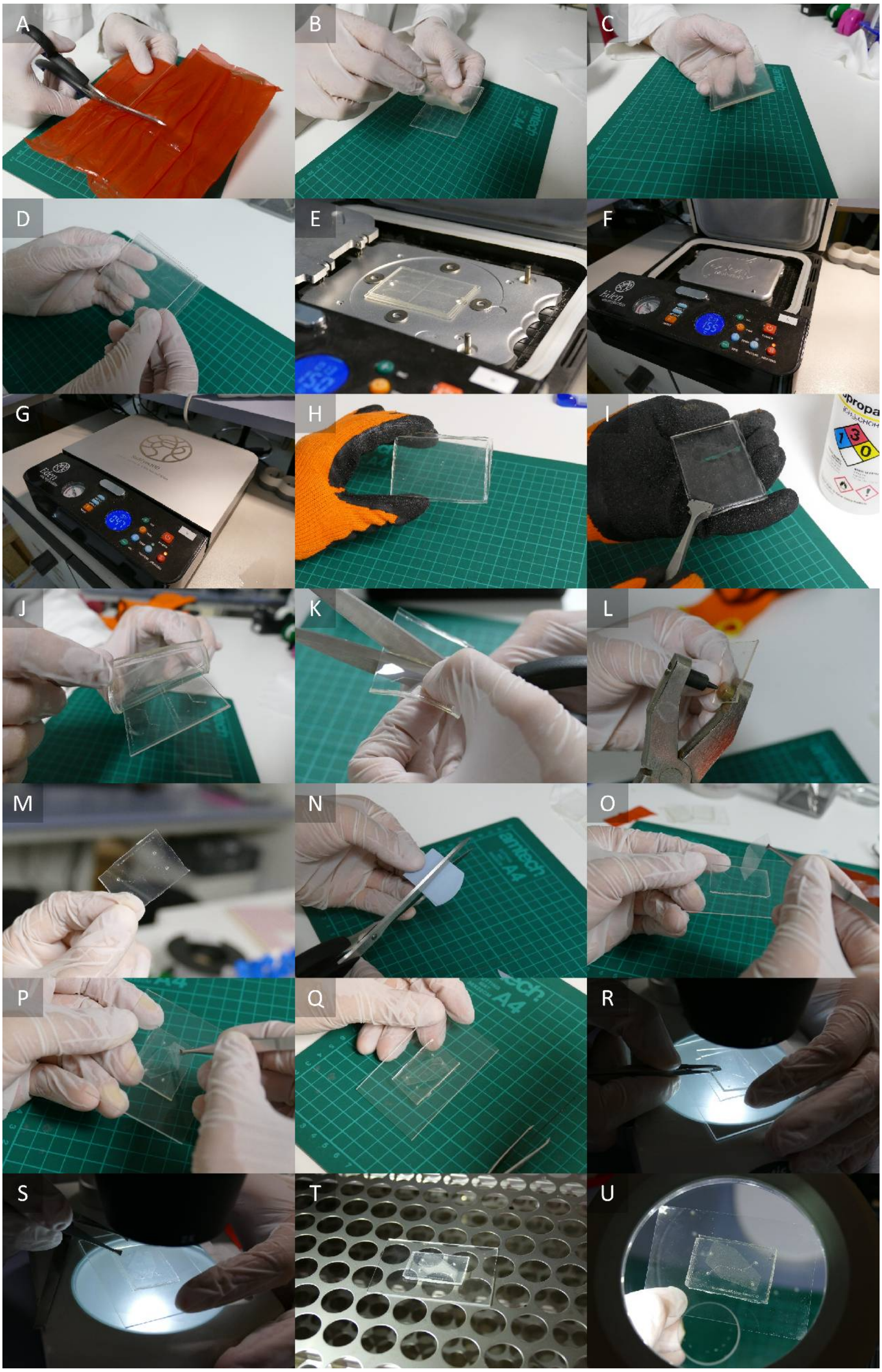
A
B
C
D
E
F
G
H
I
J
K
L
M
N
O
P
Q
R
S
T
U

Figure S1: Step-by-step fabrication procedure of a three layer, membrane-integrated sTPE-PC composite device. (A) Cutting with scissors of raw sTPE extruded sheet to approximately the size of the microfluidic mold (in this case, a glass microscope slide). Photo shows the sTPE sheet covered stored between two red Teflon films (B-C) Laying of the sTPE sheet onto the microfluidic mold, assuring maximum contact and minimal air bubbles between the sTPE and the mold. (D) Laying of a second, plain glass slide into contact with sTPE sheet to act as a stiff, flat back-plate for hot embossing. (E) Placement of the mold-sTPE-glass slide assembly onto the lower metal plate in the vacuum heat press. Image shows four spacers surrounding the assembly for final sTPE thickness control. (F) Placement of the upper metal plate to prepare the assembly for vacuum-assisted hot embossing. (G) Running of the two-minute vacuum assisted hot embossing cycle at 150˚C. (H) Mold-sTPE-glass slide assembly after removal from the vacuum heat press. (I) Removal of the glass slide back-plate from the hot embossed sTPE sheet using tweezers and isopropanol to assist with separation. (J) Removal of the hot embossed sTPE sheet from the mold. (K) Cutting with scissors of the hot embossed sTPE sheet to device-sized pieces. (L-M) Punching of access holes into the upper sTPE layer with a steel hole punch. (N) Cutting with scissors of the PC membrane to a appropriate size for the device. Note that the membrane is shown stored between blue paper films. (O-Q) Laying of the PC membrane in conformal contact with the upper sTPE layer. Light adhesion occurs immediately upon conformal contact between the PC membrane and sTPE layer. Note that the PC membrane covers the channel of the upper sTPE layer but leaves the ports accessing the channel o the lower sTPE layer unobstructed. (R-S) Manual alignment with the aid of a stereoscope of the second (lower) sTPE layer such that the central channels are superimposed and the access ports in the upper sTPE layer align with the second sTPE channel. Light adhesion occurs immediately upon contact, but is easily reversible, such that poor alignment can be corrected for. (T) Baking of the membrane-integrated device for 2 hours at 80˚C. (U) Final device after baking, ready for subsequent cell culture use.

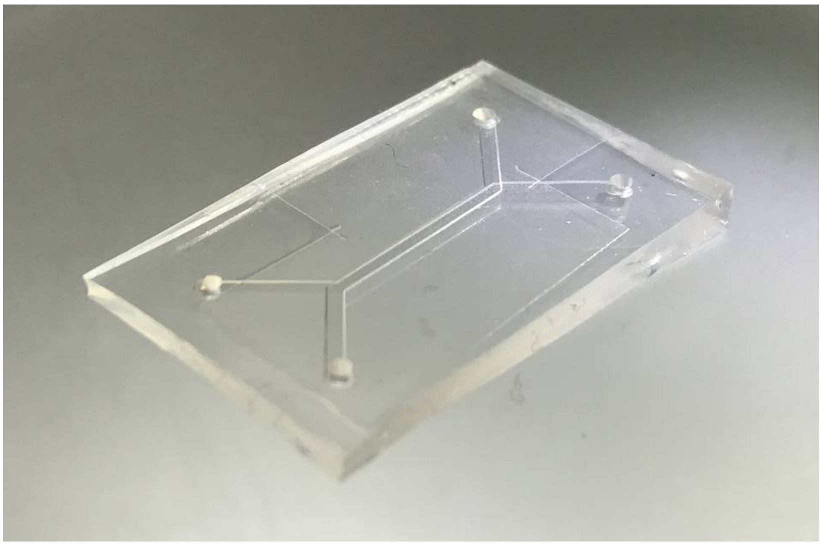

Figure S2: Composite device consisting of two micropatterned layers of Flexdym™ sTPE separated by a porous polycarbonate membrane. The two overlapping channels each measure 800 μm in width and 110 μm in thickness, and the entire device footprint is 25 mm x 35 mm.

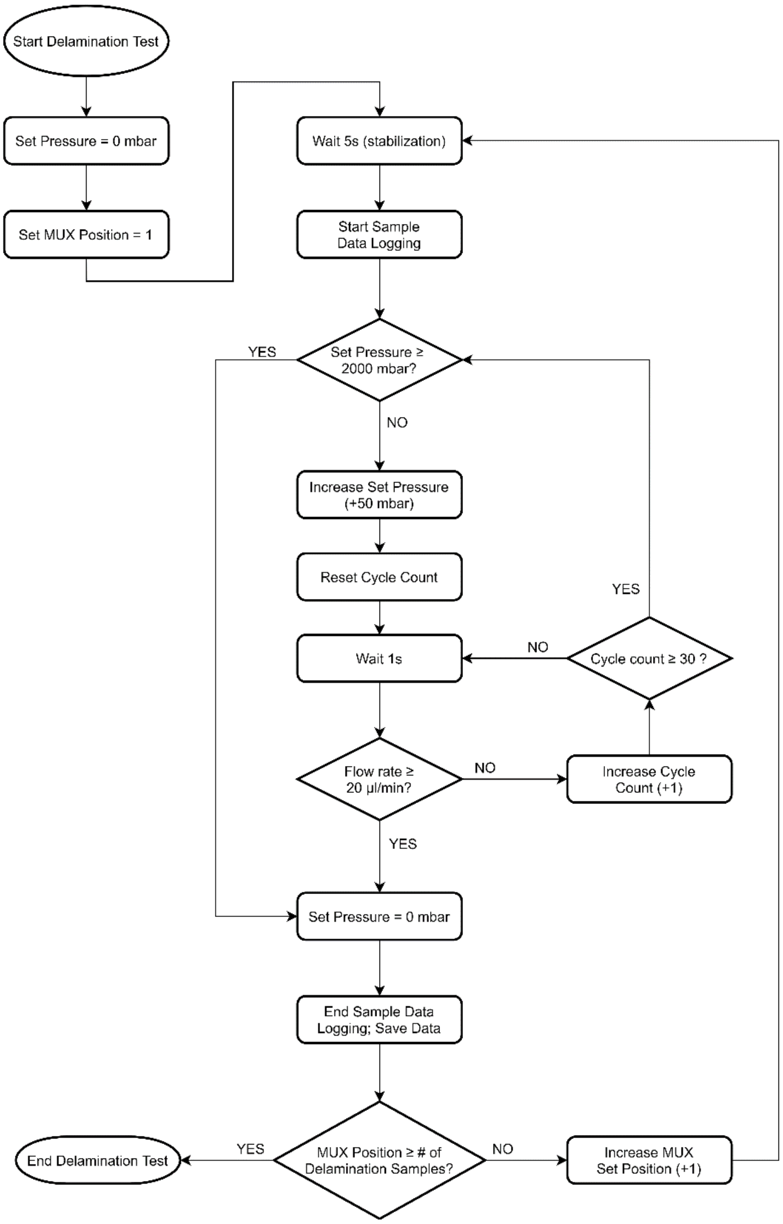

Figure S3: Logic flowchart of the automated delamination testing setup programmed in the Elveflow Smart Interface software. The sequence uses feedback from a flow sensor in order to detect device delamination (i.e. leak in the system) and stop the pressurization cycle before switching to the subsequent sample.

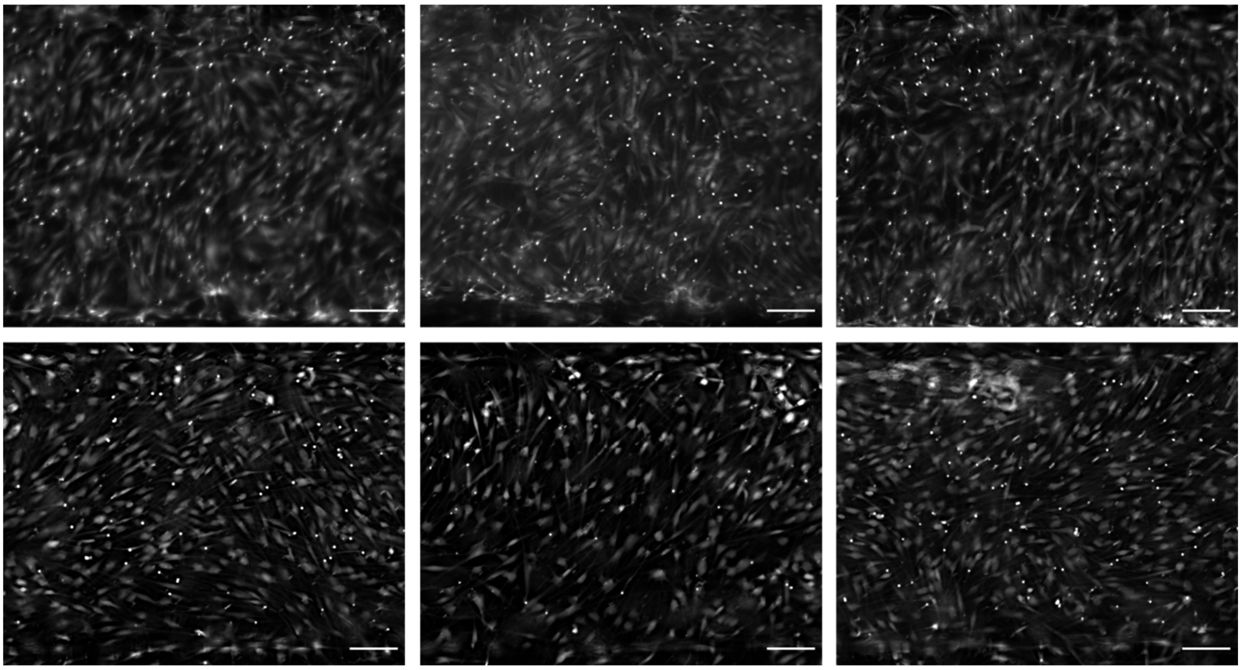

Figure S4: Human dermal fibroblasts cultured in FD-PC-FD devices. HDFs were cultured in the top layer channel, a top of the polycarbonate membrane. Cells were stained with Calcein AM in the device, prior to imaging at day 2 and day 7. Top row are cells imaged at day 2 of culturing; bottom row are cells imaged at day 7 of culturing.
